# Availability of low vision services and barriers to their provision and uptake in Ghana: practitioners' perspectives

**DOI:** 10.4314/ahs.v21i2.51

**Published:** 2021-06

**Authors:** Sylvester Kyeremeh, Khathutshelo P Mashige

**Affiliations:** Discipline of Optometry, School of Health Science, University of KwaZulu-Natal, Private Bag X54001, Durban, 4000 South Africa

**Keywords:** Low vision services, provision, barriers

## Abstract

**Background:**

Provision and uptake of low vision services are essential.

**Objective:**

To assess the availability of low vision services and barriers to their provision and uptake in the Ashanti and Brong Ahafo regions of Ghana from the perspective of eye care practitioners.

**Methods:**

A descriptive, quantitative, cross-sectional study design using semi-structured questionnaires was used to collect information from eye care practitioners selected from 58 eye care facilities in the Ashanti and Brong Ahafo regions of Ghana.

**Results:**

Forty-four eye care practitioners from Ashanti region and 10 from Brong Ahafo region responded to the questionnaire. Seventeen (34%) of the 50 eye care facilities who reported having patients seeking low vision services in their facilities provided such services. Lack of low vision devices (94.4%) and equipment (87%) were reported to be the main barriers to the provision of low vision services. Major barriers to low vision services uptake were lack of awareness (88.7%), high cost (70.4%) and social unacceptability of low vision assistive devices (59.3%).

**Conclusion:**

Lack of adequate low vision services and barriers to their provision and uptake impact negatively on efforts to prevent visual impairment and blindness in Ghana.

## Introduction

Globally, about 285 million people are visually impaired, 39 million are blind and 246 million have low vision.^1^ Majority (90%) of visually impaired people live in low-income countries.^2^ People 50 years and older represent 65% and 82% of those who live with visual impairment and blindness, respectively.^1^ A person with low vision is one who has vision impairment even after treatment and/or standard refractive correction, and has a visual acuity of less than 6/18 to light perception, or a visual field less than 10 degrees from the point of fixation, but uses, or is potentially able to use, vision for planning and/or execution of a task for which vision is essential.^3^

Low vision reduces an individual's ability to undertake vision related tasks which could result in reduced quality of life of the person, increased dependence on family and increased depressive symptoms.^4^,^5^ Notwithstanding the debilitating effects of low vision, rehabilitation services have proven to enhance functional vision, potentially benefiting 90% of patients with the condition.^6^ However, low vision services are not always available to many people who require them, particularly in low income countries.^7^ This has been attributed to several factors including lack of eye care professionals,^8^ cost of services, non-availability of the devices, fear of stigma, lack of significant improvement in vision and inability to contact patients.^9^ Other researchers have reported concurrent major health problems and patients not feeling the need for low vision rehabilitation as common reasons for not accessing low vision services.^10^

In Ghana, while studies on the prevalence of low vision and blindness have been conducted, there is a paucity of literature on the availability of low vision services and barriers to their provision and uptake. Such information is important to provide the basis for low vision care planning and improvement in Ghana.

## Methods

A descriptive, quantitative and cross-sectional study was conducted in Ashanti and Brong Ahafo regions of Ghana between January 2015 and December 2016. A semi-structured questionnaire containing questions on demographic profile of eye care practitioners, availability of low vision services and barriers to their provision and uptake was used. The questions included categorical, ordinal and open-ended questions. The design of the questionnaire was based on the review of relevant literature.^9^, ^11^,^12^,^13^ The survey was preceded by a pilot study conducted among eye care professionals who did not take part in the final study in order to critically evaluate and finalise the questionnaire. All queries from the questionnaire during the pilot study were addressed and the questionnaire adjusted accordingly before the final study was conducted. The questionnaires were hand delivered by the principal researcher and two assistant optometrists to the eye care practitioners in the 58 eye care facilities in the study areas. One eye care practitioner from each facility was selected based on their willingness to take part in the study. If there were two or more practitioners, only the head of the facility (head of low vision unit where applicable) participated in the study. In the absence of the head, any of the eye care practitioners was requested to complete the questionnaire. Follow-ups were made through telephone calls, and the completed questionnaires were collected from the practitioners by the principal researcher and the two assistant optometrists. In order to categorize a facility as providing low vision services, the eye care practitioner had to respond “yes” to the question “Do you currently offer any form of low vision services in your facility?”

Data were analysed with Statistical Package for Social Sciences (SPSS) version 20. The data from closed response questions was analysed descriptively and open response questions were summarized. Descriptive statistics such as frequencies and percentages were utilized and results presented in tables and figures. Pearson's chi-square test was used to assess statistical significance in observations. A p value less than or equal to 0.05 was considered significant. Permission to conduct the study was obtained from the Biomedical Research and Ethics Committee, University of KwaZulu-Natal (BREC Reference number: BE438/14) and the Ethical Review Committee of the Ghana Health Service (Ethical Approval ID No.: GHS-ERC: 09/10/14). Gatekeeper permission letters were obtained from the relevant authorities in each eye care facility. Eye care practitioners were provided with information documents and consent forms. Their identities remained confidential.

## Results

### Demographics

Eye care practitioners from 44 facilities in Ashanti and 10 in Brong Ahafo regions responded to the questionnaire, giving a response rate of 93%. The facilities included 35 public, 16 private and 3 non-governmental organisations. Thirty-two males and 22 females responded to the questionnaires on behalf of their facilities, and their mean age was 32.6±6.48 years (range, 26 to 54 years). The eye care practitioners included 49 optometrists, 4 ophthalmic nurses and 1 ophthalmologist. There were 19 (35.2%) facilities in Kumasi Metropolis, followed by Sunyani Municipal 4 (7.4%), Ejisu Juaben District 3 (5.6%), Bekwai Municipal 3 (5.6%), Obuasi Municipal 3 (5.6%), Atwima Nwabiagya District 2 (3.7%), Atwima Kwanwoma District 2 (3.7%), Bosomtwe District 2 (3.7%), Sekyere East District 2 (3.7%), Sekyere South District 2 (3.7%), Berekum Municipal 1 (1.9%), Wenchi Municipal 1 (1.9%), Atwima Mponua District 1 (1.9%), Kintampo North District 1 (1.9%), Jaman North District 1 (1.9%), Jaman South District 1 (1.9%), Tano South District 1 (1.9%), Mampong Municipal 1 (1.9%), Asanti Akim North District 1 (1.9%) and Offinso District 1 (1.9%).

### Eye care facilities that provided low vision services

Fifty facilities had patients seeking low vision services at different times of which 33 (66%) did not provide these services and 17 (34%) did. The 17 facilities which provided low vision services included 10 (58.8%) public, 5 (29.4%) private and 2 (11.8%) non-governmental organisations. In all these facilities, the services were provided by optometrists. Out of the 17 facilities providing low vision services, 11 (64.7%) were in Ashanti region while 6 (35.3%) were in Brong-Ahafo region.

### Low vision equipment

The most common equipment available in all 17 facilities were direct ophthalmoscopes, trial lens sets (full aperture), universal trial frames, long handle occluder with pinhole, pen torches and measuring tapes. The least available equipment were hand disc perimeter and computer software with text enlargement and voice output. The list of available low vision equipment is shown in Table 1.

**Table 1:** Low vision equipment available in eye care facilities

Equipment	Number of facilities
WHO low vision kit	5
Streak retinoscope	15
Direct ophthalmoscope	17
Lens meter	12
Trial lens set (full aperture)	17
Universal trial frames	17
Paediatric trial frames	7
Trial lens holder	6
Halberg clip	2
Long handle occluder with pinholes	17
Cross cylinders (±0.5, ±1)	17
Pen torch and measuring tape	17
**Vision assessment equipment**	
Light box for visual acuity test	7
Distant logMAR test charts-letter, number, tumbling Es, Landolt Cs (one of each type)	8
Near vision tests (calibrated for 40cm). reading acuity test (continuous text in English)	8
Symbol paediatric tests for matching and pointing (with and without crowding)	10
Preferential looking system	6
Contrast sensitivity test charts	10
PV-16 colour vision test (double set)	2
Amsler grids	10
Hand disc perimeter	2
Tangent screen	1
Optical low vision devices Spectacle magnifiers (half eyes)	4
Foldable and hand-held magnifiers with and without built-in light source	8
Stand magnifiers	4
Dome and bar magnifiers	3
Hand-held monocular telescopes	3
Filters	6
Closed Circuit Television Devices (CCTV) Colour television (20 inches)	2
Black and white hand-held CCTV magnifier	3
Full colour hand-held CCTV magnifier	3
Computer with laser printer and scanner	14
Computer software with text enlargement and voice output	1

### Types of clinical low vision services provided

Clinical low vision services provided in the 17 facilities ranged from history taking to dispensing of optical and non-optical devices. These services were grouped as “History and symptoms”, “Needs/Goal setting” and “Clinical assessment” (Table 2). There was no significant difference in the range of clinical low vision services provided between the two regions (χ2 =12.621(12), p = 0.397).

**Table 2:** Range of clinical low vision services provided

Service provided	Yes	No
**History and symptoms**		
Visual history	16	1
Ocular history	16	1
Medical history	16	1
Social history	14	3
Duration	14	3
Other disability (physical/mental)	13	4
Visual symptoms	15	2
Ocular symptoms	15	2
Medical symptoms	14	3
Social symptoms	14	3

**Needs/goal setting**	**Yes**	**No**

Distance tasks	13	4
Near tasks	14	3
Mobility	11	6
Daily living skills	10	7
Current assistive devices	11	6
Support	9	8
Treatment	9	8
Other needs	7	10

**Clinical assessment**	**Yes**	**No**
Distance visual acuity with logMAR chart	7	10
Distance visual acuity with Snellen chart	14	3
Near/reading visual acuity	13	4
Verification of distance prescription	14	3
Verification of near prescription	14	3
Retinoscopy	12	5
Distance refraction	14	3
Near refraction	14	3
Accommodation if relevant	7	10
Establishing magnification	9	8
Contrast sensitivity	5	12
Glare function	5	12
Color vision	7	10
Visual field if relevant	12	5
Low vision assistive devices	9	8
Dispensing low vision assistive devices	9	8
Training in use of low vision devices	8	9
Advice and referral if necessary	13	4

### Barriers to providing low vision services

**Figure 1:**
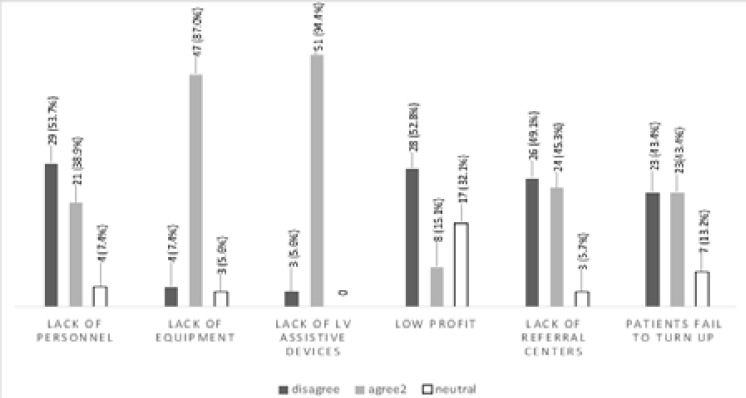
Barriers to providing low vision services

Lack of low vision devices 51 (94.4%) and equipment 47 (87%) were reported to be main barriers to the provision of low vision services. Other barriers to providing low vision services are shown in Figure 1.

### Barriers to uptake of low vision services

**Figure 2:**
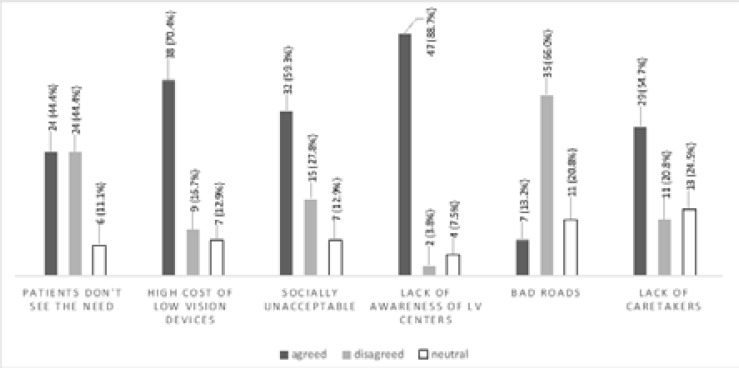
Barriers to uptake of low vision services

Practitioners were requested to report what they perceived to be barriers preventing patients from utilising low vision services. Forty-seven (88.7%), 38 (70.4%) and 32 (59.3%) practitioners reported that lack of awareness, high cost of low vision devices and socially unacceptable devices, respectively, were the main barriers to uptake of low vision services by patients. Other reported barriers are shown in Figure 2.

## Discussion

This study reports on the availability of low vision services and barriers to their provision and uptake in two regions of Ghana, from the perspective of eye care practitioners. The study showed that there is limited availability of low vision services. Lack of equipment and low vision devices were found to be major barriers to providing low vision care while unawareness of low vision centres, high costs and social unacceptability of devices were perceived as major barriers to uptake of services by low vision patients. Most common barriers reported are external to the practitioners themselves and have also been mentioned in other studies.^11^,^12^,^13^ These results have implications for planning and implementing low vision services in these regions.

Most eye care facilities were public institutions, which might be a reflection of the Ghana Health Services' aim to include eye care in many of its facilities. The majority of eye care practitioners were located in urban areas. This urban-rural disparity is similar to other health care disciplines in the country^8^. The relatively young mean age of practitioners in these regions augurs well for the profession as they will remain in active service for a longer time serving their communities. Boadi-Kusi et al^14^ reported similar young mean age of eye care practitioners in other parts of Ghana. Most practitioners (90.7%) providing low vision services were optometrists, possibly because this cadre of eye care workforce's training has more emphasis on low vision compared to other eye care professionals.^14^

Most eye care facilities reported that they had patients who required low vision services. This suggests that there is need for such services possibly among the elderly due to the increasing life expectancy rates in Ghana.^15^ It is known that prevalence of low vision increases with age.^16^ Yapa et al^17^ reported that there is demand for low vision services in Ghana but few facilities provided it.

The few eye care facilities that provided low vision services lacked many low vision-specific equipment and low vision assistive devices (Table 1 and Figure 1). Only eight facilities owned logMAR charts, which are preferable to Snellen visual acuity charts for low vision patients for the following reasons: Snellen visual acuity charts have few letters at poorer acuity levels (only one at 6/60) which can make a person with low vision feel dispirited. Snellen acuity charts display unequal number of optotypes on each line and unequal increments between lines. Each of these factors impacts the accuracy of visual acuity and magnification measurements. As a result of unequal optotypes on each line, patients with better vision are exposed to more letters to read which can result in ‘crowding’. Considering these characteristics, visual acuity at different distances cannot be equated. LogMAR acuity charts have standard increments and optotypes making them more appropriate when testing low vision patients.^18^,^19^

Most tests performed were part of routine eye examination. Tests that have important role in clinical low vision assessment such as contrast sensitivity and glare testing were not routinely performed. Contrast sensitivity gives better assessment of the patient's ability to function in the real world and it's useful when considering the utility of low vision devices and when to refer someone who has lens opacities as secondary pathology.^20^ Colour vision was not routinely assessed, possibly because not much can be done to improve it. Visual field testing was reported to be performed if indicated, probably because this is more of a function than disease detection or monitoring test (usually, disease causing the vision loss is known).

### Barriers to the provision of low vision services

Practitioners indicated that lack of equipment (87%) and low vision devices (94%) were major barriers to the provision of low vision services (Figure 1). This may be attributed to the general non-availability of these equipment and devices on the Ghanaian market and high import duties. Other researchers^9^,^11^–^13^ also identified lack of low vision devices as a barrier to providing this service. Appropriate equipment is necessary to provide comprehensive low vision services.

Lack of referral centers and patients' failure to turn up for care were other barriers to the provision of low vision services. Lack of information about referral centers among eye care professionals could result in failure to refer, thereby, negatively affecting uptake of low vision services. Effective communication and liaisons among eye care professionals might help publicize existing referral centers and encourage practitioners to refer cases when necessary. Jose et al^13^ also found lack of availability of low vision care centers as major barriers to low vision care in India. There could be many reasons why patients fail to turn up for low vision services. O'Connor et al^21^ identified proximity and convenience as the main facilitators to service use while issues concerning transport, needing an accompanying person, lack of information about the service and poor health were the main barriers in their study in Melbourne, Australia.

### Barriers to the uptake of low vision services

Practitioners reported that “unawareness of low vision centres” was the highest impediment to patients utilising low vision services. This finding supports the reports from other studies that lack of public awareness about low vision care is a significant barrier to uptake of low vision services.^12^,^13^ Okoye et al^12^ reported that lack of public awareness of low vision care was a major barrier to clinical low vision provision in Nigeria. These results suggest the need to raise awareness on patients' general health and eye care issues, which could in turn increase low vision service uptake. Exploring appropriate advertising avenues and organizing outreach programs to inform the public about low vision and its care centers may help to enhance awareness among patients and improve uptake of these services.

High cost of low vision devices was also reported to be a major barrier to patients using low vision services. Adjusting pricing system of devices could accommodate the purchasing capacity of majority in the community, and address the burden of low vision. Many people with low vision in the regions studied rely on government disability benefits and pension as a primary source of income. Therefore, their ability to afford the high cost of low vision devices is a cause for concern. In addition to cost and affordability, it would be useful to understand aspects of “lack of need” and “social unacceptability” in greater depth, by probing, for instance, the social and cultural factors that lead to someone feeling this way with regards to low vision devices.

Other issues related to uptake of services are influenced by the dynamics in the family, culture and community. Lack of caretakers/caregivers has been reported as a barrier to uptake of services by low vision patients. Marmamula et al^22^ reported that “individuals who cannot draw upon the support of family or care givers to accompany them to the clinic or to provide related assistance may be less likely to act upon a need when it is felt”.

## Limitations

This study has certain limitations. Firstly, this was a quantitative study and is therefore subject to all the shortcomings of quantitative studies, including limited in-depth understanding of the participants' responses. Secondly, the results of this study are based on practitioners' responses from two regions of Ghana and cannot be generalized to all the practitioners in the country.

## Conclusion

The study showed that availability of low vision services is limited in Ashanti and Brong Ahafo regions of Ghana. Barriers to the provision of low vision services reported by the practitioners are mainly economic barriers such as lack of equipment and assistive devices. These barriers can be addressed with the service providers, by making the equipment available and devices affordable. Barriers to uptake of low vision services reported are relatively more challenging to change or address. Lack of ‘felt need,’ awareness of low vision centres and personal reasons such as social unacceptability of the devices and lack of caretakers/caregivers/supportive family structure, require a sustained long-term effort both at the individual level and by the service providers to create an impact. Understanding and addressing these barriers are essential to planning low vision services in these regions.
